# Observations on the Pathogenesis of Sarcoma 37 Mouse Ascites Tumour

**DOI:** 10.1038/bjc.1957.14

**Published:** 1957-03

**Authors:** P. Warner, H. Kroeker, J. M. Lederman

## Abstract

**Images:**


### OBSERVATIONS ON THE PATHOGENESIS OF SARCOMA 37 MOUSE ASCITES TUMOUR

P. WARNER,* H. KROEKER AND J. M. LEDERMAN

*From the Department of Pathology, Medical School, University of Manitoba, Winnipeg, Canada*

Received for publication January 7, 1957

TRANSPLANTABLE mouse ascites tumours provide a useful experimental tool for the application of quantitative methods to the study of cancer. However it is important to be aware of the conditions under which cells multiply in such tumours and of possible differences existing between them and, for example, " solid " subcutaneous tumours.

It has been suggested that the cells of ascites tumours multiply while freely suspended in peritoneal fluid. Another proposition would be that inoculated cells multiply within tissues in microscopic foci to produce daughter cells that are liberated from the foci to provide an increasing number in the peritoneal fluid. If the first hypothesis is correct then cells in suspension in ascitic fluid are actively metabolizing and dividing. Whereas, if the second hypothesis is true the cells in ascitic fluid may not be in a state of active metabolism and division ; if this is so, the chemical and other properties of suspended cells may be different from those of cells in actively growing tumour tissue. It is clear then that, before drawing far reaching conclusions from experiments on ascites tumours, the metabolic condition of the constituent cells should be known.

Goldie and Felix (1951) concluded that the cells of the ascites form of Sarcoma 37 divided while in suspension in peritoneal fluid because they could detect an increase in number of cells in the fluid before vascularization could have taken place. Klein (1951) investigating the same tumour came to the same conclusion because he detected a similar increase in tumour cells in mice in which he could find no histological evidence of tumourous infiltration of intra-abdominal tissues. Therefore it is thought important to present the following contrary observation that growth of Sarcoma 37 ascites tumours takes place in tissue before an increase of cell number occurs in peritoneal fluid. In addition no entirely satisfactory explanation for the mechanism of accumulation of ascitic fluid following the intraperitoneal inoculation of ascites tumour cells has been presented. Observations providing some indication of how ascitic fluid accumulates under these conditions will be described.

Klein's (1951) definition of ascites tumours is such that it assumes multiplication of freely suspended cells. For this communication his definition is unsuitable. Transplantable mouse ascites tumours will be regarded as ones which have produced ascites containing tumour cells such that if small quantities of the fluid so produced are inoculated intraperitoneally into other healthy mice, peritoneal fluid containing tumour cells will accumulate.

* Present address : The Institute of Medical and Veterinary Science, Adelaide, Australia.

This paper is divided into two parts—the first consists of general observations on the later stages of development of the 7th and 8th passages of an ascites tumour obtained from a strain of subcutaneously transplanted Sarcoma 37. The second part describes experiments on the early stages of development of the same tumour in the 9th to the 12th passages. The experiments indicate the mechanism of accumulation of ascitic fluid and support the contention that ascites tumour cells multiply in intra-abdominal fatty tissues before the occurrence of an increase of cells and fluid in the peritoneal cavity.

### MATERIALS AND METHODS

*Sarcoma 37.*—Mice bearing subcutaneous tumours of Sarcoma 37 were received from Dr. Franks of the Banting Institute, Toronto. The tumours were removed, cut finely with scissors, ground in a pestle and mortar and made into an approximately 5 per cent suspension in saline. Volumes of 0·2 ml. were injected intraperitoneally into mice. When abdominal swelling occurred, in this and subsequent passages, the peritoneal cavity was opened, fluid removed and injected intraperitoneally into further mice in volumes ranging from 0·05 to 0·5 ml. In the 7th and 8th passages, which are described in the " General Observations " section, 31 mice were inoculated with from 1·3 to 10·4 million tumours cells. The mice were killed 5 to 10 days after inoculation.

*Mice.*—Connaught strain white mice were used throughout. In the 1st to the 8th passages 6 to 10 week old mice were used and the sexes were segregated. From the 9th to the 12th passages only males from 6 to 8 weeks of age were used.

*Cell counts.*—Tumour cells were counted in a haemocytometer. Ascitic fluid was diluted 1 in 20 in saline containing methyl violet. Tumour cells were recognized by their large size and by the large, usually irregular nucleus and by the almost invariable presence of spherical shining granules in the cytoplasm.

*Measurement of the volume of ascitic fluid.*—Volumes of ascitic fluid greater than 2·0 ml. were measured in a 10-ml. measuring cylinder. Either a 0·2-ml. pipette graduated in 0·001 ml. or a 1·0-ml. pipette graduated in 0·01 ml. was used for smaller volumes. The point of the pipette was placed in the interstices of the intestines and run over the peritoneal surfaces so that as much fluid as possible was collected. In normal male mice aged from 6 to 10 weeks it was not possible to obtain more than 0·05 ml. of fluid.

*Histological sections.*—From mice described in the " General Observations " section, tissues were taken as indicated by naked eye observation at post-portem. From each mouse described in the " Experimental " sections the entire pancreatic area and associated peritoneal folds were dissected out. Both of the fat bodies attached to the testes and a piece of anterior abdominal wall, excluding skin and subcutaneous tissues were removed. The tissues were fixed in Helly's fluid and sections prepared in the usual way were stained with haematoxylin and eosin. As will be mentioned in the appropriate part of the results, serial sections were cut from many of the blocks and every tenth was stained and examined.

### GENERAL OBSERVATIONS

### *Results*

All 31 mice examined had excess fluid in the peritoneal cavity ranging from 0·25 to 8·0 ml. in volume. Counts were performed on the fluids of 14 mice. In 13 the total number of tumour cells was increased over those inoculated. The ascitic fluid from the mouse in which there was no increase of cells was haemorrhagic, contained clumps of tumour cells and " soft tumour " (to be described below). Smears revealed a large proportion of inflammatory and other non-tumour cells, sometimes greater than that of the tumour cells.

" *Soft tumour* ".—The most remarkable and frequent observation was the presence of a whitish-yellow opaque mass in or protruding from the region lying caudal to the liver and stomach and bounded on either side by the spleen and duodenum. Gentle dissection revealed that these masses lay among the peritoneal folds that contained the pancreas. The masses were soft and friable and occurred in 19 of the 31 mice-examined. Occasionally (in 3 out of 31 mice) similar masses were seen in the substance of the bodies of fat attached to the testes ; here they were seen as yellowish opaque areas contrasting with the greyish white translucency of the normal fat. Masses of a similar appearance and consistency were frequently seen lying free in the peritoneal cavities of mice that had been inoculated with large numbers of cells or where a long time had elapsed since inoculation with smaller numbers.

Histological examination of these masses which are referred to as " soft tumour ", showed that the greater part of them was completely avascular and consisted of rounded cells with a pinkish blue opaque cytoplasm and darkly staining nuclei ; mitoses were not infrequent. These were compared with those appearing in the substance of tissues. The latter were approximately oval or polygonal in shape, with a comparatively transparent and delicately basophilic cytoplasm. The nucleus was large and vesicular. In fact the cells of " soft tumour " resembled those in ascitic fluid more closely than those apparently growing in tissues. Fig. 1 shows soft tumour from the pancreatic area lying adjacent to growth occurring in and replacing fat ; the difference between the two types of cell may be seen.

Occasionally, in sections of " soft tumour " lying free in the peritoneal cavity, there was a small vascular core of tumour tissue in the centre of the mass of rounded cells. The periphery of some of those " soft tumours " taken from the pancreatic area was bounded by peritoneum. The " soft tumours " occurring in the gonadal fat bodies were slightly different in their histological picture in that they appeared to consist of an aggregation of rounded cells within oedematous interstitial tissue.

*Growth in fatty tissue.*—The second observation of interest was the invariable appearance of tumour growth in histological sections of intra-abdominal fatty tissue. Sections were made from the fatty tissue of 22 mice and in every one vascular tumour could be seen. Most of the sections were taken from the region of the pancreas which, in the mouse, consists of separate lobes invested by two layers of peritoneum. In between the lobes of the pancreas the two layers of peritoneum fuse as one membrane to be separated in places by blood vessels and islands of fat invisible to the naked eye. Some sections were made from the bodies of fat attached to the gonads. At their point of attachment these bodies are of small circumference but towards their free ends they enlarge and split up into a number of processes separated by deep clefts.

Growth in the fatty tissue (Fig. 2) appeared to be diffuse, replacing fat cells, but did not seem to extend beyond the limits of the region originally occupied with fat. Cells beyond those limits were rounded and not vascularized and did not appear to take part in the tumour formation within the fat (Fig. 3). Sometimes the growth in the fat bodies appeared to spread by direct continunity along the visceral peritoneal membranes (Fig. 4), occasionally apparently replacing vessel walls (Fig. 3). The distinction between the visceral peritoneal growth and that occurring on the parietal peritoneum (described below) should be noted.

*Peripancreatic oedema.*—On one occasion the pancreas was seen to be surrounded by a relatively large quantity of crystal clear gelatinous material. Histological section showed this to consist of markedly oedematous peripancreatic tissue limited peripherally by peritoneum (Fig. 5). Subsequently the pancreatic area was examined for the presence of oedema which was seen fairly commonly. Sometimes in the oedematous peripancreatic tissues numerous tumour cells were

---

EXPLANATION OF PLATES

FIG. 1.—34/55–5 12. Section of "soft tumour" in the pancreatic region of a mouse 5 days after inoculation with 6·4 million tumour cells. On the left tumour may be seen replacing fatty tissue. The remains of a few fat cells are shown. Towards the right the cells are rounded and not organized as vascular tumour. Cell nuclei become progressively smaller and darker and the cytoplasm more readily seem towards the right. × 85.

FIG. 2.—24/55–4 9. Section of peritoneal fat from a mouse 5 days after inoculation with 10·4 million tumour cells. Permeation between and replacement of fat cells is seen together with sheets of tumour tissue. × 85.

FIG. 3.—39/55–11 6. Section of peritoneal fat from a mouse 46 hours after inoculation with 3·1 million tumour cells, showing replacement of fatty tissue with tumour cells. There is also an intense infiltration with inflammatory cells. Rounded cells can be seen at the margin of the tissues and at the lower left hand corner is a vessel whose wall is replaced by tumour cells. × 145.

FIG. 4.—24/55–2 8. Section of fat and peritoneal membranes from a mouse 5 days after inoculation with 10·4 million tumour cells. Small islands of fat are shown replaced by tumour growth which extends along the intervening peritoneal membranes. × 85.

FIG. 5.—38/55–10 4. Section of pancreas from a mouse 6 days after inoculation with 6·6 million tumour cells. The peripancreatic tissues are markedly oedematous. No tumour cells are to be seen. The peritoneal surface is at the top. × 42.

FIG. 6.—30/55–17 7. Section of pancreas from a mouse 5 days after inoculation with 3·1 million tumour cells. Tumour and inflammatory cells are shown in the oedematous peripancreatic tissues. The tumour cells are of the rounded type and not part of organized growth. × 210.

FIG. 7.—24/55–4 9. Section of parietal peritoneum from a mouse 5 days after inoculation with 10·4 million tumour cells. Heavy infiltration of the muscle with tumour and inflammatory cells is shown. Note the marked vascular dilatation. The white line running transversely across the upper part represents the original peritoneal surface on which there is a thick vascular layer of tumour and inflammatory cells. An occasional rounded cell is seen on the surface. × 85.

FIG. 9.—39/55–1 (2) 11. Section of peritoneal fat from a mouse 6 hours after inoculation with 3·1 million tumour cells. A single tumour cell is shown within the tissue applied to a fat cell wall. There is also marked vascular dilatation and infiltration with inflammatory cells. × 425.

FIG. 10.—39/55–1 (1) 10. Section of peritoneal fat from a mouse 6 hours after inoculation with 3·1 million tumour cells. Dense infiltration with inflammatory cells is shown with marked vascular dilatation. Among the inflammatory cells are larger ones which may be tumour cells. There are one or two rounded cells at the periphery of the fat. × 425.

FIG. 11.—39/55–3 (1) 1. Section of peritoneal fat from a mouse 9 hours after inoculation with 3·1 million tumour cells. Shows a dense inflammatory focus at the tip of one of the processes of a testicular fat body. To the right of the focus there is a single tumour cell lying between three fat cells. × 300.

FIG. 12.—40/55–1. Section of peritoneal fat from a mouse 18 hours after inoculation with 3·1 million cells. Shows part of a testicular fat body in which there is most of a large and part of a smaller area of interstitial oedema occupied by rounded tumour cells. Within the tissue itself there are numerous tumour cells lying between the fat cells. In the upper right portion there is a small group of tumour cells. At the top on the left dilated capillaries may be seen, one with a tumour cell applied to it. × 150.

1

2

3

4

5

6

Warner, Kroeker and Lederman.

7

9

10

11

12

seen (Fig. 6). The cells were of the rounded type seen in ascitic fluid and "soft tumours". No blood vessels were seen in association with them. Such collections of cells were not considered to constitute tumour growth.

*Parietal peritoneal growth.*—On occasions the parietal peritoneum showed a roughened pebbly appearance. Sections revealed thin layers of vascularized growth occurring on and in the parietal peritoneum (Fig. 7). Although tumour growth frequently appeared in the anterior abdominal wall at the site of inoculation it was not observed to spread in the parietal peritoneum from that site.

Fourteen sections of the spleen and 10 of the liver were taken from 14 mice with ascites during the 1st to the 12th passages none of them showed growth within or on the surface of the organs.

### EXPERIMENTAL I

As a result of the general observations which were made on relatively advanced ascites tumours, experiments were planned to study the early development to see what relationship the findings had to the appearance of an increase of fluid and cells in the peritoneal cavity.

### *Method*

Four experiments were carried out in the 9th to the 12th passages in which 70 mice were inoculated with from 3·1 to 6·6 million tumour cells. After inoculation mice were killed at intervals varying from 6 hours to 7 days. Post-mortems were performed on the 70 mice and the amount of peritoneal fluid measured. Histological sections were examined for the presence of tumour cells in both testicular and peripancreatic tissues, peripancreatic oedema and tumour growth on the parietal peritoneum. Of a possible total of 280 examinations, 14 were not carried out because specimens were either lost or inadequate.

### *Results*

The results are shown in Tables I and II and illustrated in Fig. 8.

*Growth in fatty tissue.*—From the tables it may be seen that histological examination revealed tumour cells within fatty tissue in every mouse including those in which there was no increase of peritoneal fluid. Fig. 8 shows that it was the earliest phenomenon to be detected. In the early stages when tumour cells were single or in small groups, they were recognized and differentiated from normal normal cellular constituents by their very large size, clear basophilic cytoplasm and large vesicular nucleus. They were not rounded but approximately oval or polygonal in shape. Some could be seen applied to blood vessels, some to fat cells and some were in mitosis. As time went on single tumour cells first became more numerous and then appeared in groups replacing fat cells and finally as vascular sheets of growth. Often, where growth occurred in the testicular fat bodies, there were nearby regions of interstitial oedema in which rounded tumour cells were present.

*Peripancreatic oedema.*—Peripancreatic oedema was seen in sections from some mice killed during the earliest period and frequently in mice without excess peritoneal fluid. Fig. 8 shows that on the average it occurred after the detection of tumour cells in fat and before the appearance of ascites. With the exception of 3 mice peripancreatic oedema was always present when there was excess fluid

TABLE I.—*A Comparison of the Times of Appearance of Tumour Growth, Peripancreatic Oedema and Ascites in Mice Inoculated with Sarcoma 37 Ascites Tumour*

| Time after Inoculation (hours) | Tumour cells in fat | Peri-pancreatic oedema | 0·1 ml. or more of ascitic fluid | Parietal peritoneal growth |
|---|---|---|---|---|
| 0–24 | 15/15 | 4/15 | 0/15 | 0/15 |
| 25–48 | 13/13 | 10/13 | 2/13 | 0/13 |
| 49–72 | 11/11 | 11/11 | 7/11 | 5/11 |
| 76–168 | 31/31 | 24/26 | 31/31 | 22/29 |
| Total | 70/70 | 49/65 | 40/70 | 27/68 |

Numerators = number of mice affected.
Denominators = number of mice observed.

TABLE II.—*The Relation between Ascites Production and the Appearance of Tumour Growth and Peripancreatic Oedema in Mice Inoculated with Sarcoma 37 Ascites Tumour*

| Relation to appearance of 0·1 ml. or more of ascitic fluid | Tumour cells in fat | | Peri-pancreatic oedema | Parietal peritoneal growth |
|---|---|---|---|---|
| | Pancreatic area | Testicular bodies | | |
| Before | 29/29 | 29/30 | 17/30 | 1/30 |
| After | 35/35 | 39/39 | 32/35 | 26/38 |
| Total | 64/64 | 68/69 | 49/65 | 27/68 |

Numerators = Number of mice affected.
Denominators = Number of mice observed.

in the peritoneal cavity. The 3 exceptions occurred in mice with small amounts of ascitic fluid of 0·25 ml. or less.

In oedematous peripancreatic tissue, rounded cells were scanty or absent in mice killed in the early stages. As time went on they became more and more numerous. At no time were collections of these rounded cells seen to be vascularized.

*Parietal peritoneal growth.*—Infiltrating vascularized tumour growth on the parietal peritoneum occurred only after 48 hours (Table I) and, with one exception, was always accompanied by excess peritoneal fluid (Table II). Thus, in general, growth on the parietal peritoneum occurred after the production of ascitic fluid (Fig. 8).

*Soft tumour.*—In this series of animals " soft tumour " was never seen before the 4th day nor did it appear in the testicular fat bodies. On the 4th day 3 out of 12 animals were affected: one had " soft tumour " in the pan-

FIG. 8.—Graphic representation of data from Table I. The sequence of changes occurring in the development of Sarcoma 37 ascites tumour.

Abscissa : Time in hours after inoculation.
Ordinate : Proportion of mice showing :
(a) Tumour cells in fatty tissue. ●————●
(b) Peripancreatic oedema. ⊙— — — —⊙
(c) 0·1 ml. or more of peritoneal fluid. ×—·—·—×
(d) Parietal peritoneal growth. +- - - - - - - -+

creatic region only and one had it lying free. The other mouse had it in both sites. After the 4th day, 17 out of 19 mice were affected. Ten had " soft tumour " in the pancreatic region only, 2 had it lying free and 5 had it in both places. The ascitic fluid of those mice with " soft tumour " was usually haemorrhagic. In 9 of the 10 counts performed the total number of tumour cells in mice with " soft tumour " was markedly lower than that in fluids from mice killed earlier and sometimes was less than the number inoculated.

### EXPERIMENTAL II

The mice in one of the experiments described above were examined in greater detail and the salient features have already been reported in brief (Warner, 1955).

### *Method*

Twenty-one mice were inoculated with 3·1 million sarcoma cells and groups of them were killed during varying periods after inoculation. In addition to the investigations described in " Experimental I " cell counts were performed on each peritoneal fluid.

### *Results*

The results are shown in Table III. During the first 22 hours there is no significant increase in the total number of tumour cells over those inoculated and no accumulation of peritoneal fluid. In fact, since it was possible to collect barely enough fluid to provide for that (0·02 ml.) required for the tumour cell count, it is possible that there was a diminution in the total number of tumour cells over those inoculated. However in each mouse killed during this period tumour cells were detected in sections of intra-abdominal fatty tissue.

Tumour cells were scanty in sections from mice killed between 6 and 10 hours and sometimes could only be seen (Fig. 9) after prolonged examination of 20 to 30 sections of each tissue. The earlier sections showed inflammatory cells in dense foci in which vascular dilatation was marked (Fig. 10). It was noticed that when they were scanty tumour cells could be seen clearly only near or at the edge of the foci (Fig. 11). Within the inflammatory foci, cells, which were thought to be tumour cells, were seen but, owing to the dense infiltration of inflammatory cells, they were not sufficiently distinct for their nature to be determined for sure (Fig. 10). In mice killed between 18 and 22 hours tumour cells were more numerous sometimes occurring in small groups.

TABLE III.—*The Relation between Increase of Cells and Fluid and the Appearance of Tumour Growth and Peripancreatic Oedema in Mice Inoculated with Sarcoma 37 Ascites Tumour*

| Time after inoculation (hours) | Mouse number | Tumour cells (millions per ml.) | Volume ascitic fluid (ml.) | Total tumour cells (millions) | Average tumour cells (millions) | Tumour cells in fat | Peri-pancreatic oedema | Parietal peritoneal growth |
|---|---|---|---|---|---|---|---|---|
| 6–10 | 1 | 33·4 | | <3·3 | | + | 0 | 0 |
| | 2 | 28·1 | | <2·8 | | + | 0 | 0 |
| | 3 | 24·2 | <0·1 | <2·4 | <2·9 | + | 0 | 0 |
| | 4 | 32·3 | | <3·2 | | + | 0 | 0 |
| | 5 | 28·5 | | <2·9 | | + | 0 | 0 |
| 18–22 | 6 | 24·4 | | <2·4 | | + | + | 0 |
| | 7 | 24·2 | <0·1 | <2·4 | <2·5 | + | + | 0 |
| | 8 | 30·3 | | <3·0 | | + | 0 | 0 |
| | 9 | 22·3 | | <2·2 | | + | 0 | 0 |
| 30–48 | 10 | 48·4 | | <4·8 | | + | + | 0 |
| | 11 | 36·2 | <0·1 | <3·6 | | + | 0 | 0 |
| | 12 | 53·0 | | <5·3 | | + | + | 0 |
| | 13 | 46·5 | 0·25 | 11·6 | <6·8 | + | 0 | 0 |
| | 14 | 69·4 | 0·1 | 6·9 | | + | + | 0 |
| | 15 | 84·8 | <0·1 | <8·5 | | + | + | 0 |
| 72 | 16 | 118·7 | 0·1 | 11·9 | | + | + | 0 |
| | 17 | 47·8 | 0·2 | 9·6 | 11·2 | + | + | + |
| | 18 | 40·4 | 0·3 | 12·1 | | + | + | + |
| 96 | 19 | 28·9 | 0·3 | 8·7 | | + | + | + |
| | 20 | 33·0 | 0·6 | 19·8 | 10·0 | + | + | + |
| | 21* | 5·4 | 0·3 | 1·6 | | + | + | + |

+ = Present.
0 = Absent.
* = This mouse has soft tumour lying free in the peritoneal cavity.
All mice were inoculated intraperitoneally with 3·1 million tumour cells.

Between 30 and 48 hours there was probably, on the average, an increase in the total number of tumour cells over those inoculated. In sections of fatty tissue from all mice killed during this period groups of sarcoma cells were present and small vascular sheets of them replaced fatty tissue.

At 72 and 96 hours there was a definite increase of cells and peritoneal fluid. However in mouse 21 the total number of cells was less than the number inoculated ; this mouse had soft tumour lying free in the peritoneal cavity. Sections from all mice killed during this period showed vascular sheets of sarcoma replacing relatively large areas of fatty tissue, similar to those shown in Fig. 2.

### DISCUSSION

The most important part of the present investigation is that tumour cells were found in intraperitoneal fatty tissue before an appreciable increase of cells or fluid occurred in the peritoneal cavity of mice inoculated with Sarcoma 37 ascites tumour. Therefore it is important to consider two points in the techniques used in arriving at this conclusion.

As far as the measurement of small amounts of fluid is concerned it was only possible to obtain quantities less than 0·1 ml. from normal male mice, it was considered sufficiently accurate for the purpose to regard volumes of 0·1 ml. or more as a criterion of increased production of fluid. Using this criterion it was found that on the average, fluid increase was correlated with the increase in total number of tumour cells (Table III). This finding supported our view as it is in keeping with that of other workers (Klein and Revesz, 1953 ; Patt and Blackford, 1954).

The other important point to be considered is the recognition of single tumour cells. This is notably difficult, but they could be seen clearly in the sparsely nucleated fatty tissue and were felt to be sufficiently distinctive to diagnose confidently as tumour cells. In any event there was no difficulty in recognizing foci of tumour cells at a later stage. It was felt that the presence of foci was compatible with the finding of single cells at an earlier stage.

As a result of these considerations and the fact that all mice with ascites showed clearly recognizable tumour in fatty tissue, it was concluded that the growth of this ascites tumour took place in tissues before an increase of tumour cells and fluid occurred in the peritoneal cavity. In addition since growth was not observed on or in the liver and spleen and only at a later stage on the parietal peritoneum, it was concluded that early invasion of fatty tissue was an invariable characteristic of this tumour. Furthermore our observations are compatible with the hypothesis that tumour cells are liberated from growth foci in fatty tissue and pass into the peritoneal cavity. Hence, growth foci in tissues could be the sole source of the increasing number of tumour cells in suspension in peritoneal fluid.

The results show that tumour cell infiltration of fatty tissue was followed by peripancreatic oedema which preceded the appearance of ascitic fluid. It is reasonable to conclude that tumour growth in the region of the pancreas causes oedema by interfering with the local vascular supply. This is supported by the observation that tumour cells replace vessel walls. The oedema fluid thus produced may be assumed to pass into the peritoneal cavity thereby producing ascites. The occurrence of oedema in the testicular fat bodies under similar circumstances suggests that the same sequence of events may occur there. It is also possible that the inflammatory reaction occurring soon after the inoculation of tumour cells, through markedly dilated blood vessels (Fig. 9, 10 and 11), contributes to the ascites. At a later stage when the peritoneum is widely involved with vascular tumour tissue (Fig. 7), fluid may pass from its altered surface into the cavity. Although it is not contended that asciitic fluid collects by one of these mechanisms only it seems most likely that in the early stages the main contribution is from the peripancreatic area where interference with the main radicles of the portal vascular system is particularly liable to produce ascites.

No progressively enlarging solid tumour growth comparable to that occurring in subcutaneously transplanted Sarcoma 37 was observed within the peritoneal cavities of mice used in this investigation. Where tumour growth occurred in fat it replaced normal tissue but did not extend as vascular tumour beyond its confines. It was concluded that "soft tumours" were merely aggregates of, free tumour cells liberated from foci of growth in fatty tissue and, in many instances, trapped by peritoneal folds. Aggregation of cells would diminish the number in suspension and thus explain the low tumour cell counts obtained when " soft tumour " was present. " Soft tumours " lying free in the peritoneal cavity could have been aggregates of cells formerly in suspension, but some of them may have been detached from the pancreatic region which could explain the presence of vascular cores in them and the concomitant haemorrhage. Observation on a phenomenon similar to " soft tumour " occurring in the Ehrlich ascites tumour are described by Bailliff (1954) who uses the term " semi-solid " tumour.

It follows from our conclusions that it is not necessary to assume multiplication of Sarcoma 37 cells while they are in suspension in peritoneal fluid. This conflicts with the views of Goldie and Felix (1951) and Klein (1951) ; it may be that these findings apply only to Sarcoma 37 or the strain of mice used ; or, it may be claimed that in twelve passages the tumour had not become adapted to the intraperitoneal environment sufficiently well for the cells to multiply in peritoneal fluid ; or the findings could be attributed to the number of tumour cells inoculated being too low to produce typical ascites tumours. Against these objections it may be said that Sarcoma 37 is not a discriminating tumour and it grows readily in many strains of white mice. Also the number of passages and tumour cells inoculated are both in excess of those used by Goldie and Felix (1951) to obtain the ascites form of the same tumour.

On *a priori* grounds one would expect what is in fact a graft of tumour cells to behave in the peritoneal cavity in approximately the same way as in subcutaneous tissues. Warner, Gostling and Thackray (1950) have provided evidence that the majority of cells in a subcutaneous graft of Sarcoma 37 becomes necrotic ; a few cells survive and pass from the graft into the host tissues where they multiply in the neighbourhood of blood vessels from which, presumably, they obtain the necessary nutriment. The findings described in this paper suggest that a similar sequence of events may occur in the ascites form of the same tumour. Thus, the formation of new vessels is unnecessary for the growth of the tumour because the cells find their way to the neighbourhood of already existing vessels. Hence the claim of Goldie and Felix (1951) that they could detect an increase of Sarcoma 37 ascites tumour cells in peritoneal fluid before vascularization could have taken place is not sufficient to support their view that multiplication takes place while the cells are in suspension.

In contrast to the findings described in this paper, Lasnitzki (1953) using Sarcoma 37 ascites tumour concluded that round free cells grew more rapidly than spindle cells that were morphologically similar to those occurring in tissues. She found that, when free ascites tumour cells were embedded in a plasma clot, at first they remained round but after incubation for 24 hours they became spindle shaped. Subcutaneous grafts in mice of these spindle cells produced fewer tumours and grew more slowly than grafts of round cells. However, the spindle cells had been incubated for 24 hours whereas the round cells had not and this circumstance alone may have accounted for the slower growth of spindle cell grafts. In further support of her contention, Lasnitzki (1953) states that, in plasma clot, mitoses were seen in round cells only. However, the presence of mitotic figures in cells does not necessarily mean that they are actively dividing. A mitotic figure may only indicate the state of a cell at the moment of its removal from an environment suitable for growth. While the cell is in suspension or otherwise removed from a suitable environment, it may remain in a state of suspended animation or dormancy in which the mitotic figure remains stationary. Furthermore it has never been shown that " free " or suspended cells will increase in number in stationary tissue culture and will only do so under special conditions in moving cultures. It is a common observation, during the initiation of cultures on glass of such tissue as HeLa cells, monkey kidney tissue and human amnion, that the inoculated cells are rounded. The rounded cells become applied to the glass surface and spread out becoming spindle shaped or polymorphous. In such cultures, if the cells remain round they do not thrive, whereas spindle cells increase in number. For growth in tissue culture, cells apparently need to become attached to some support and require adequate nutriment. That similar conditions obtain *in vivo* is suggested by Lasnitzki's (1953) observations on histological preparations of subcutaneous grafts. The central portion of her grafts, which eventually became vascular tumour tissue, consisted of spindle cells. At first no growth was detected in these cells which could, by analogy with tissue culture, be regarded as being attached to a supporting structure. At a later stage, when blood vessels appeared (presumably carrying adequate nutriment), growth of the spindle cells occurred. It is easy to see that a similar explanation can be applied to the development of the ascites form of the same tumour.

At present the only crucial finding supporting a hypothesis that multiplication of tumour cells takes place in suspension would be that in which there is an increase in the number of cells combined with the absence of growth in tissues. The ability to detect tissue invasion depends on the intensity of the search. Where tumour foci in tissues are few and small in size or possibly, as in this work, obscured by inflammatory cells, the probability of detecting one may be small. In fact it would be necessary to examine serial sections of each piece of tissue before one could say with confidence that tumour growth was absent. Dealing with this matter in connection with Sarcoma 37 ascites tumour, Klein (1951) states that he detected an increase of cells in the peritoneal fluid before he could find tumour cell infiltration of tissues. He does not indicate how many sections were taken from each tissue, nor how many tissues from each mouse, nor is there clear correlation between tumour cell counts of individual mice and histological findings. Thus it is impossible to judge the intensity of his search for foci of tumour growth. Therefore there remains the possibility that an insufficient number of sections were examined to reveal early tumour growth in tissues; consequently his results are not necessarily in conflict with those described here.

If the arguments put forward are correct then it is possible that the findings described here may have a more general application to other ascites tumours. The finding of growth of the Ehrlich (Bailliff, 1954; Klein, 1951) and the Krebs (Krebs, Thordarson and Harbo, 1942) ascites tumours in fat and the description of something akin to " soft tumour " in the Ehrlich ascites tumour (Bailliff, 1954) indicate similarity between these and the Sarcoma 37 ascites tumour. Even if tissue growth is demonstrated before the occurrence of an increase in the number of cells in the peritoneal fluid in ascites tumours in general, there would still remain the question of whether any cell multiplication at all takes place in peritoneal fluid. Such findings as an exponential increase in the number of cells in suspension or changes in mitotic rate can be explained by assuming that cells are liberated from growing foci in tissues. In fact all the described phenonomena exhibited by ascites tumours can be satisfied by the hypothesis that growth occurs only in tissues, and the production of ascitic fluid can be more satisfactorily explained by this hypothesis than by that of growth in suspension. Thus, to conclude, it is unnecessary to assume that ascites tumour cells multiply in suspension in peritoneal fluid.

### SUMMARY

This paper describes observations on 101 Connaught strain white mice after inoculation with Sarcoma 37 ascites tumour in the 7th to the 12th passages.

Post-mortem examinations of 31 mice with well-developed ascites tumours showed no tumours similar to the subcutaneous form but soft avascular masses of aggregated tumour cells frequently occurred. Histological sections of intra-abdominal fatty tissue from 22 mice all showed diffuse invasion with tumour tissue.

Seventy mice were killed at intervals from 6 hours to 7 days after inoculation with 3·1 to 6·6 million tumour cells. Sections of fatty tissue from all mice showed the presence of tumour cells ; 30 of the mice had not developed appreciable ascitic fluid. Tumour cell counts were performed in 9 mice within 22 hours of inoculation and there was no increase in number over those inoculated. Seventeen out of 30 mice without appreciable ascites and 32 out of 35 with ascitic fluid showed peripancreatic oedema. The three mice in the latter group had only 0·25 ml. or less of ascitic fluid. Parietal peritoneal growth occurred relatively late in the development of ascites tumours and was observed only once in 30 mice without ascitic fluid and in 26 out of 38 mice with ascitic fluid. Aggregated tumour cells were present in some mice from the 4th day onwards.

From the results it was concluded that growth of Sarcoma 37 ascites tumour occurred in fatty tissues before an appreciable increase of cells or fluid in the peritoneal cavity. Growth in peripancreatic fatty tissue interfered with the portal circulation causing oedema which contributed fluid to the ascites. Foci of growth liberated cells into the peritoneal fluid. Liberated cells often become aggregated to form tumour-like masses but no true tumour formation took place. In view of these results it is unnecessary to assume that ascites tumour cells multiply within peritoneal fluid.

The authors are indebted to Miss L. Nason for the preparation of the photomicrographs and to Mr. G. A. Gormly for the preparation of Fig. 8. This work was supported by a grant from the National Cancer Institute of Canada.

### REFERENCES

BAILLIF, R. N.—(1954) *Cancer Res.*, **14**, 554.
GOLDIE, H. AND FELIX, MARIE D.—(1951) *Ibid.*, **11**, 73.
KLEIN, G.—(1951) *Exp. Cell Res.*, **2**, 291.
*Idem* AND REVESZ, L.—(1953) *J. nat. Cancer Inst.*, **14**, 229.
KREBS, C., THORDARSON, O. AND HARBO, J.—(1942) *Acta Radiol., Stockh., Suppl.*, **44**, 1.
LASNITZKI, I.—(1953) *Brit. J. Cancer*, **7**, 238.
PATT, H. M. AND BLACKFORD, MARGARET E.—(1954) *Cancer Res.*, **14**, 391.
WARNER, P.—(1955) *Nature*, **176**, 1030.
WARNER, P. T. J. C. P., GOSTLING, J. V. T. AND THACKRAY, A. C.—(1950) *Brit. J. Cancer*, **4**, 396.


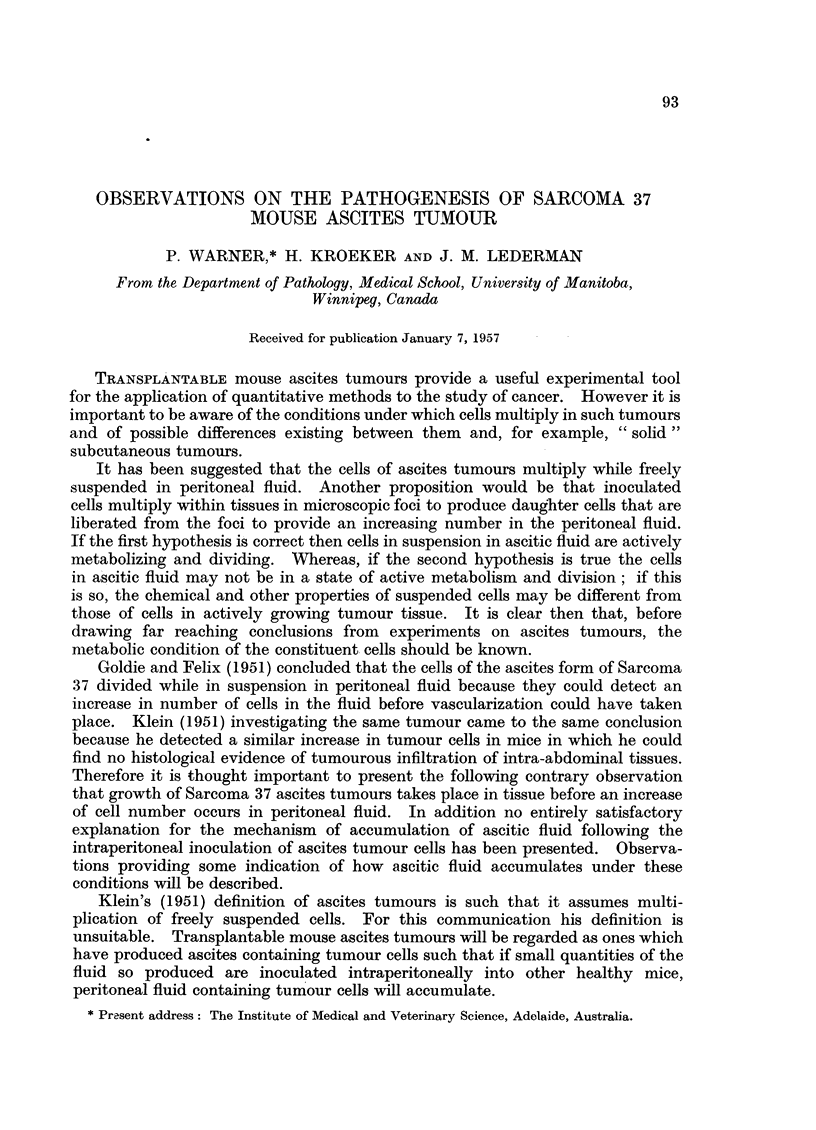

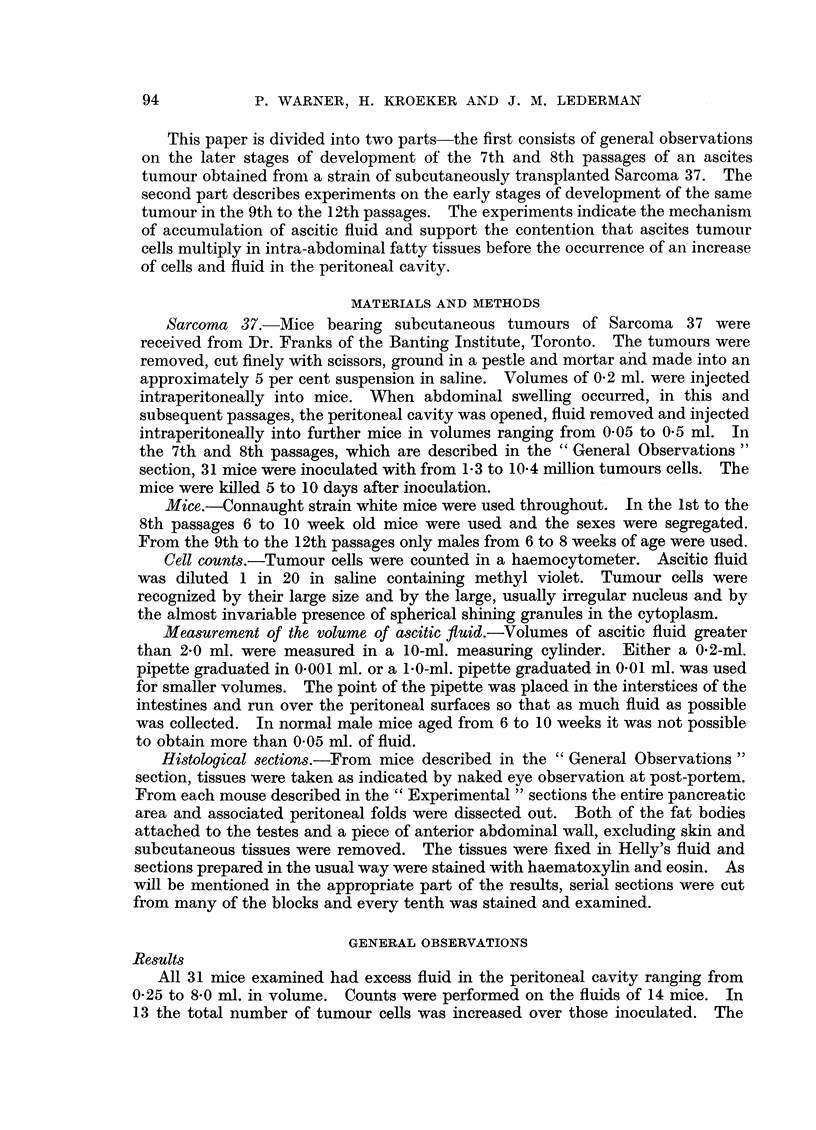

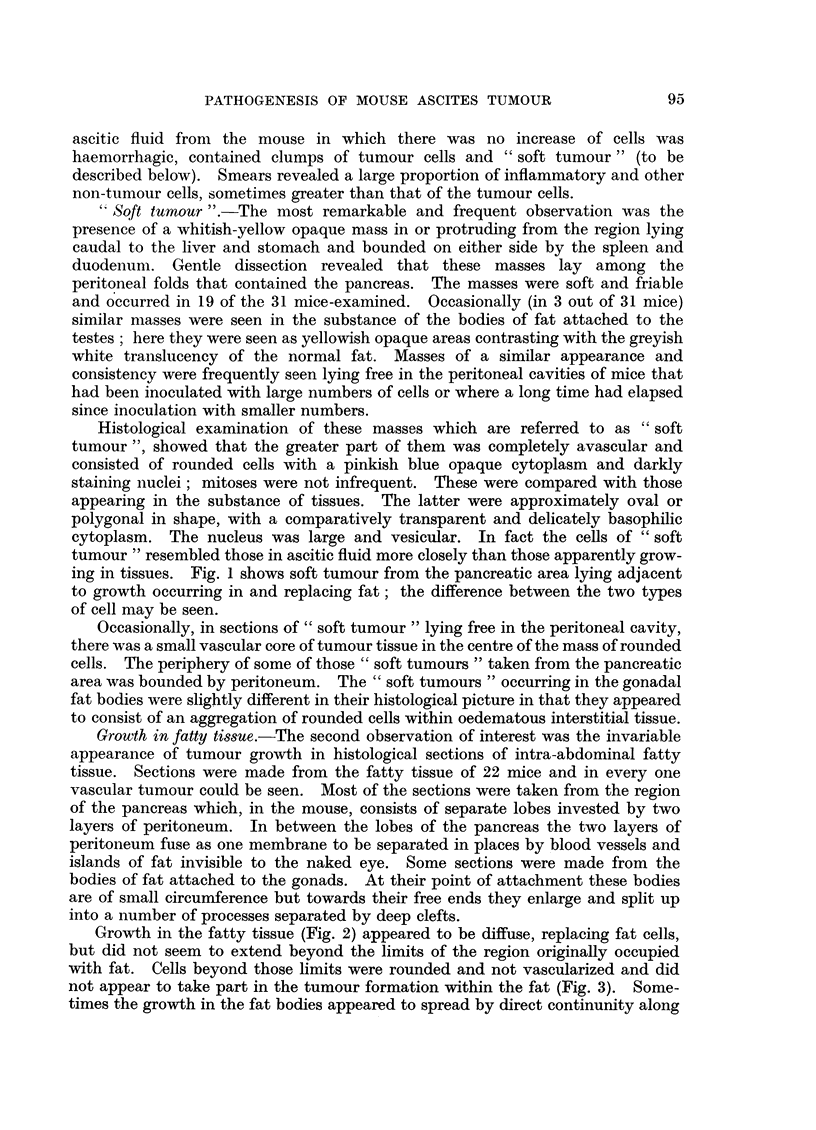

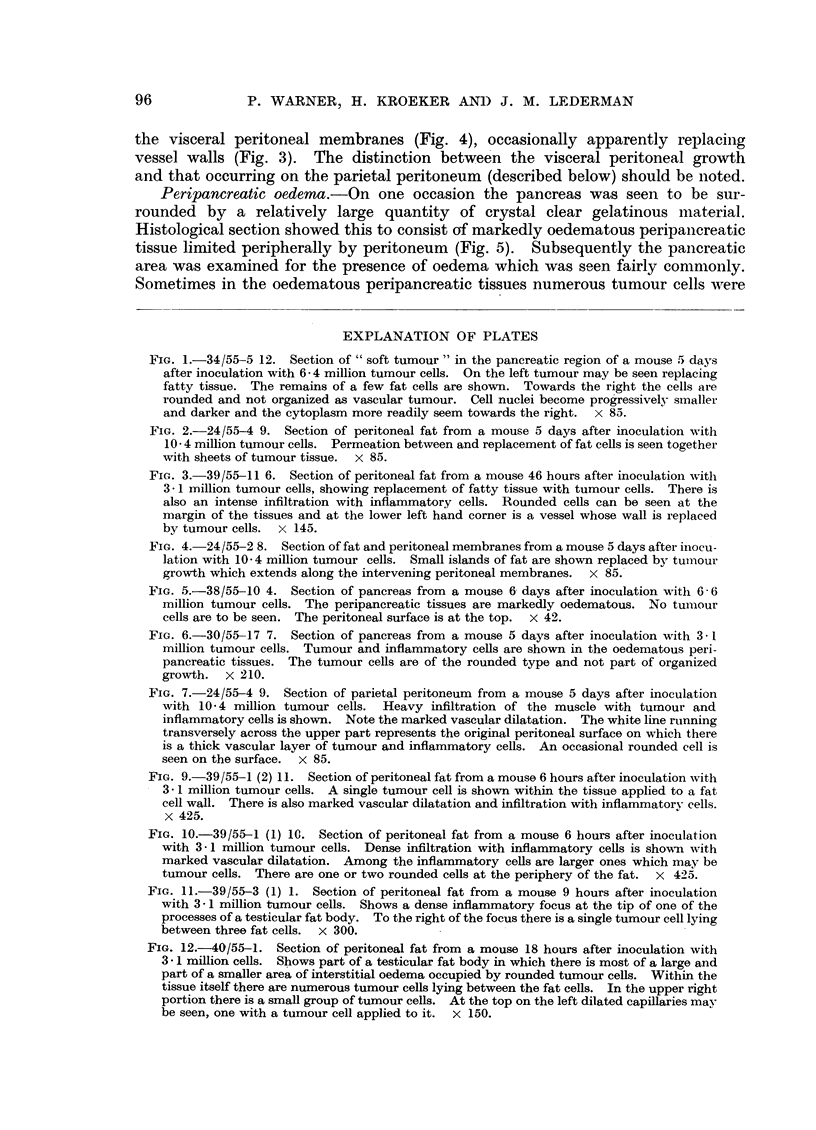

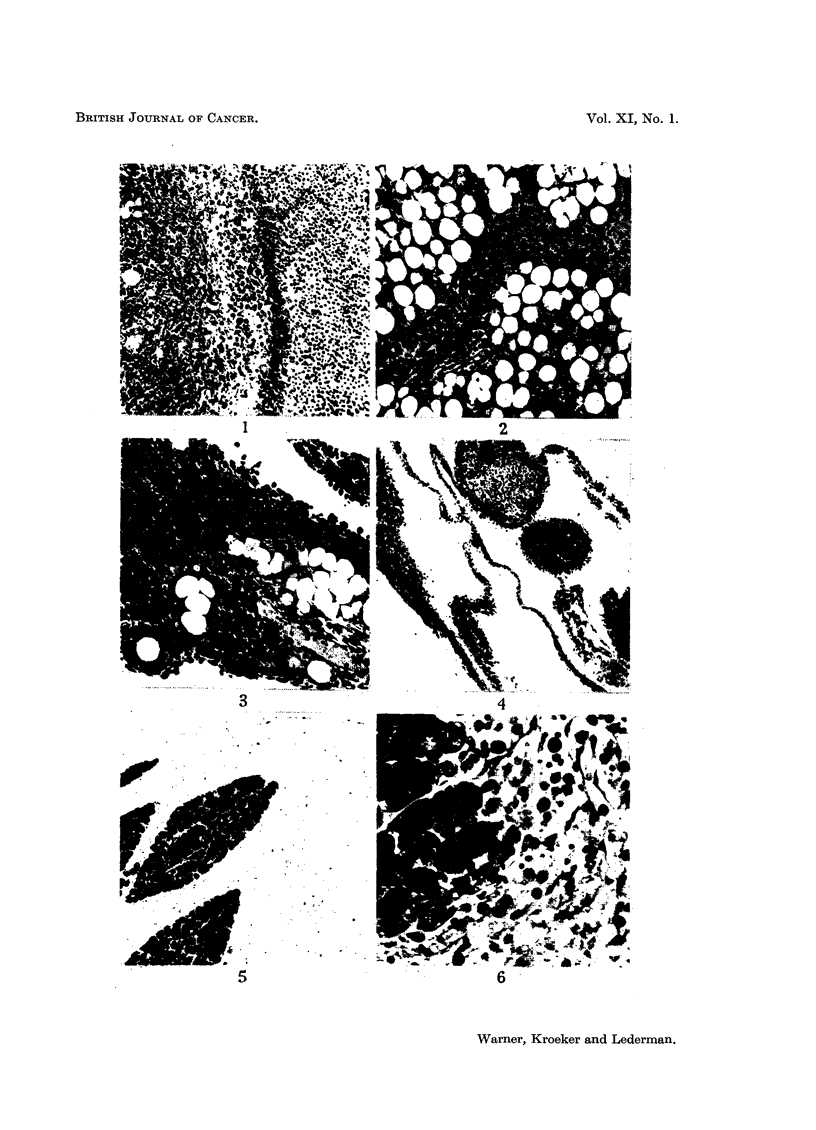

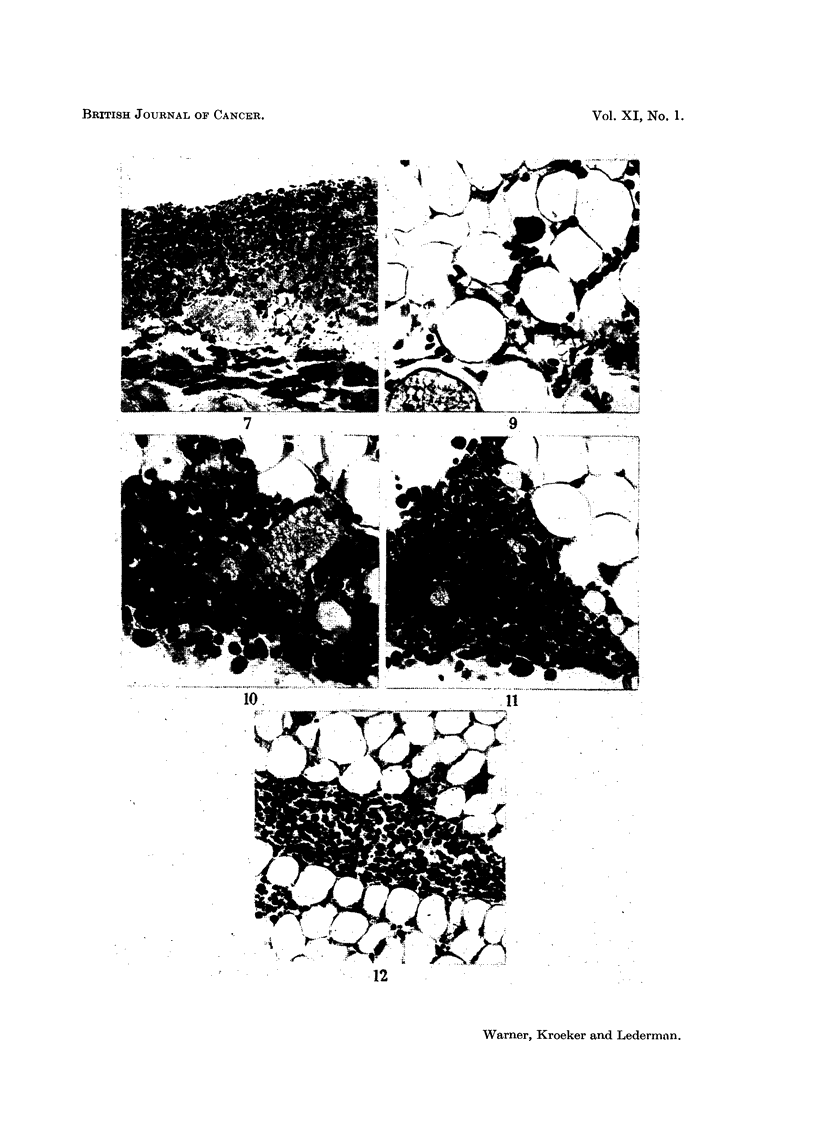

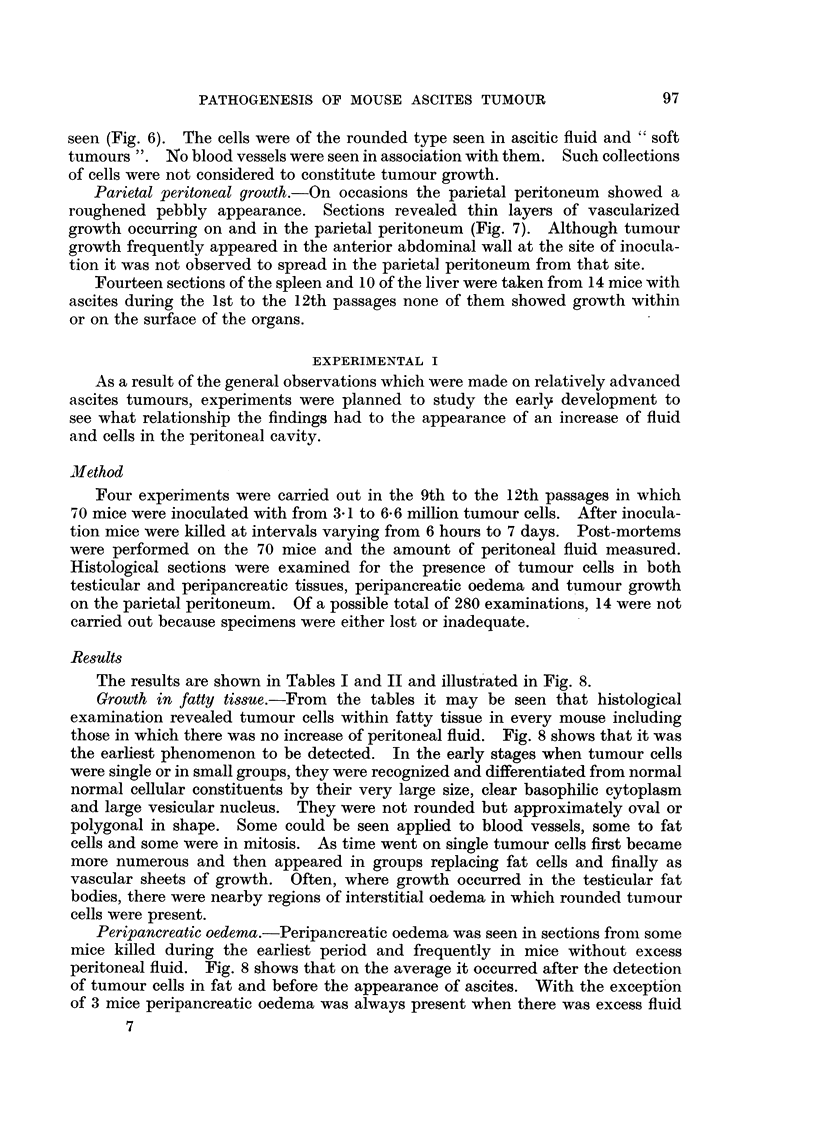

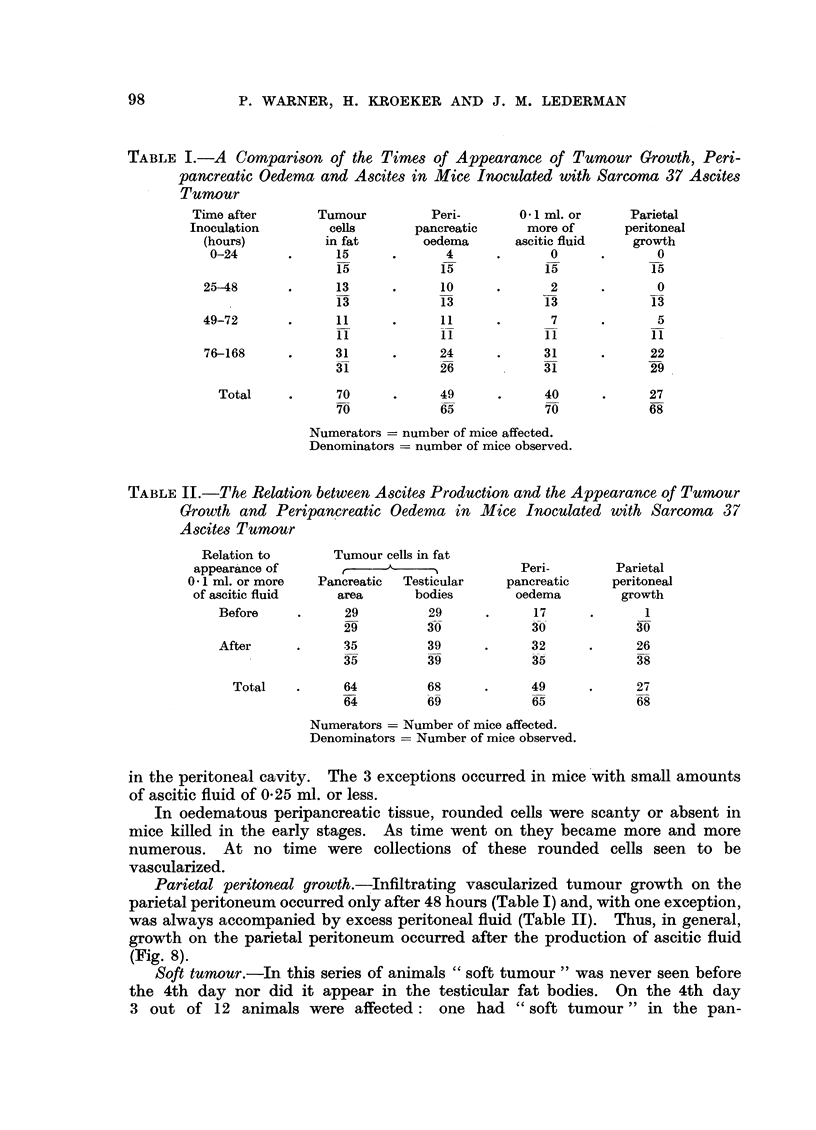

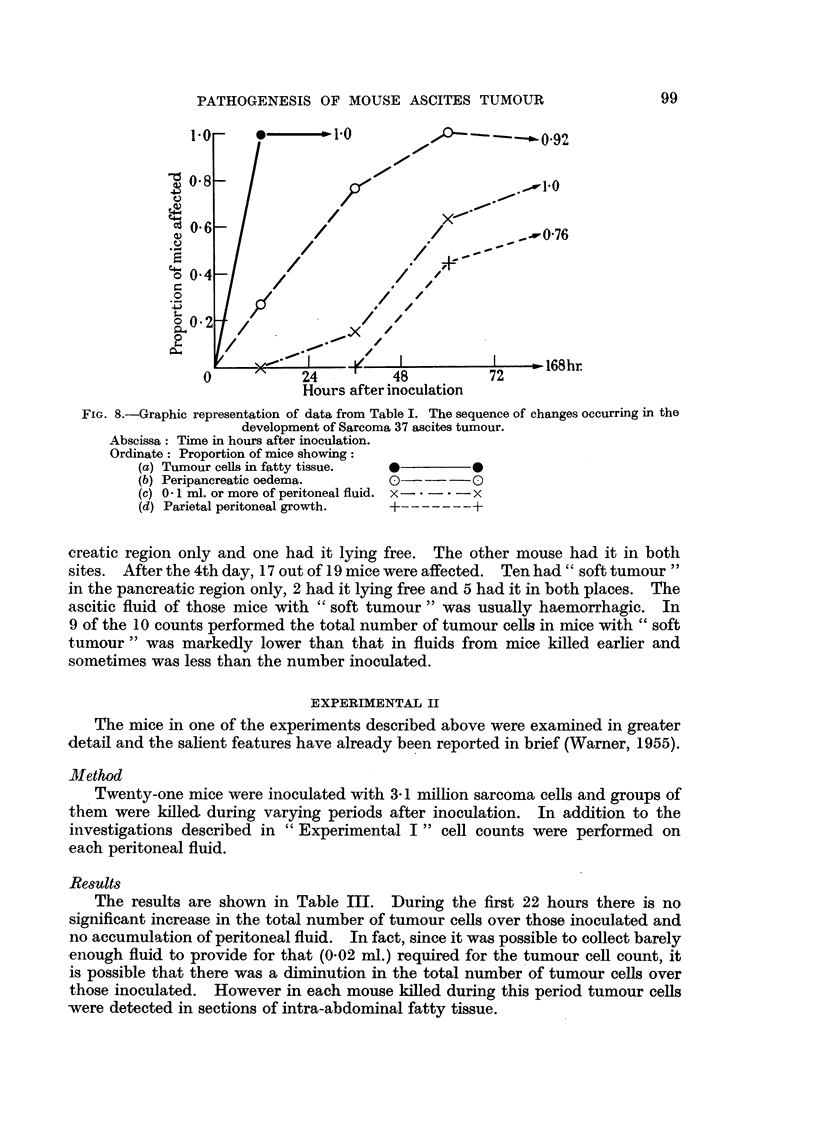

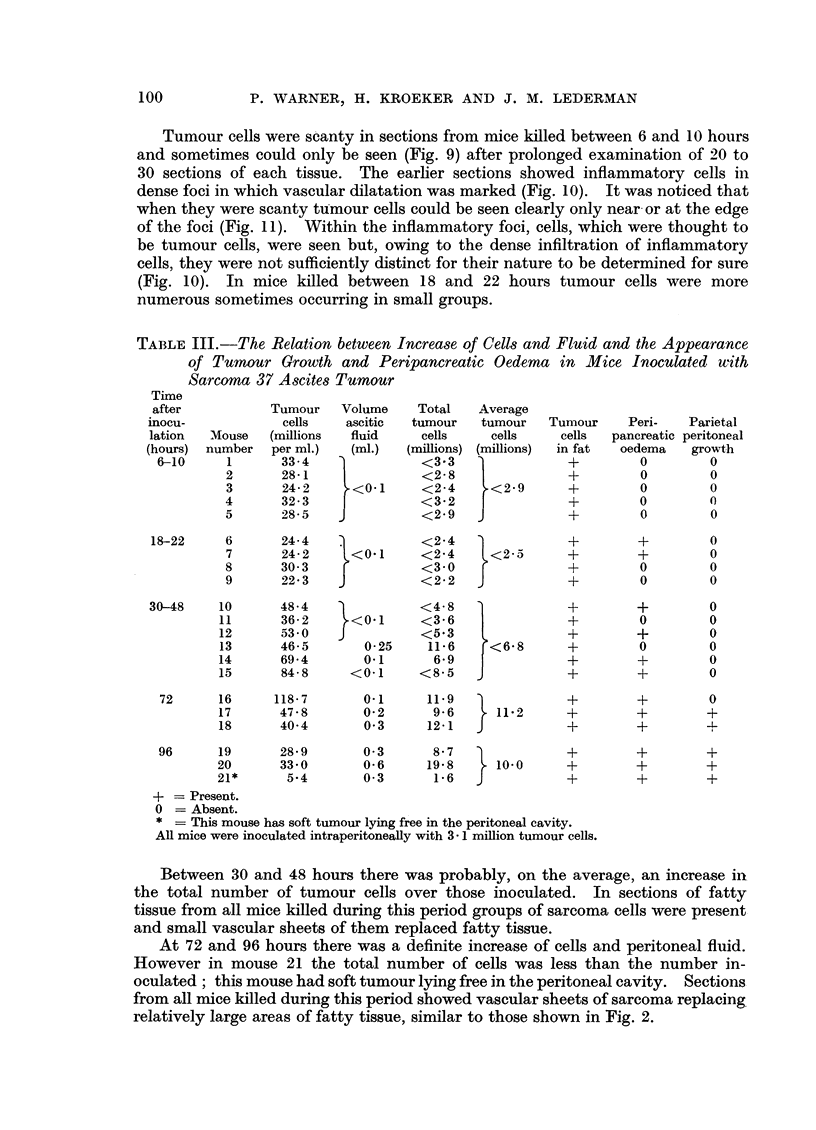

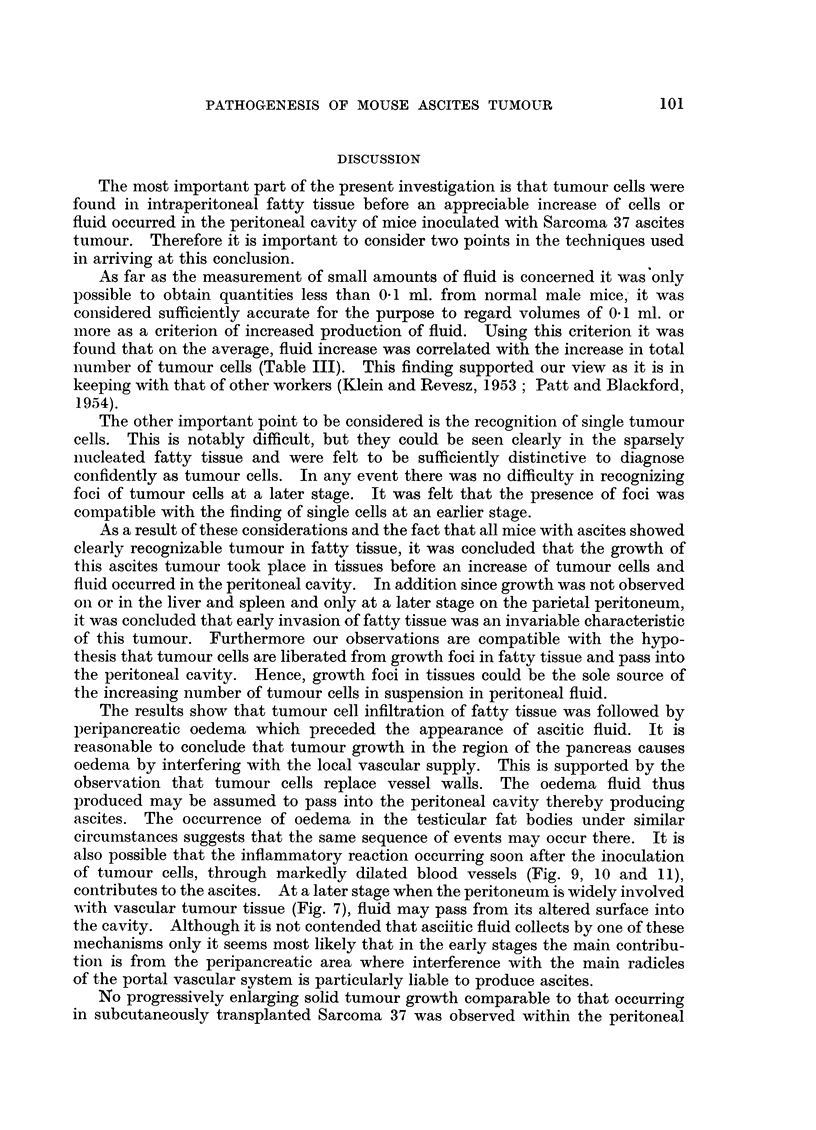

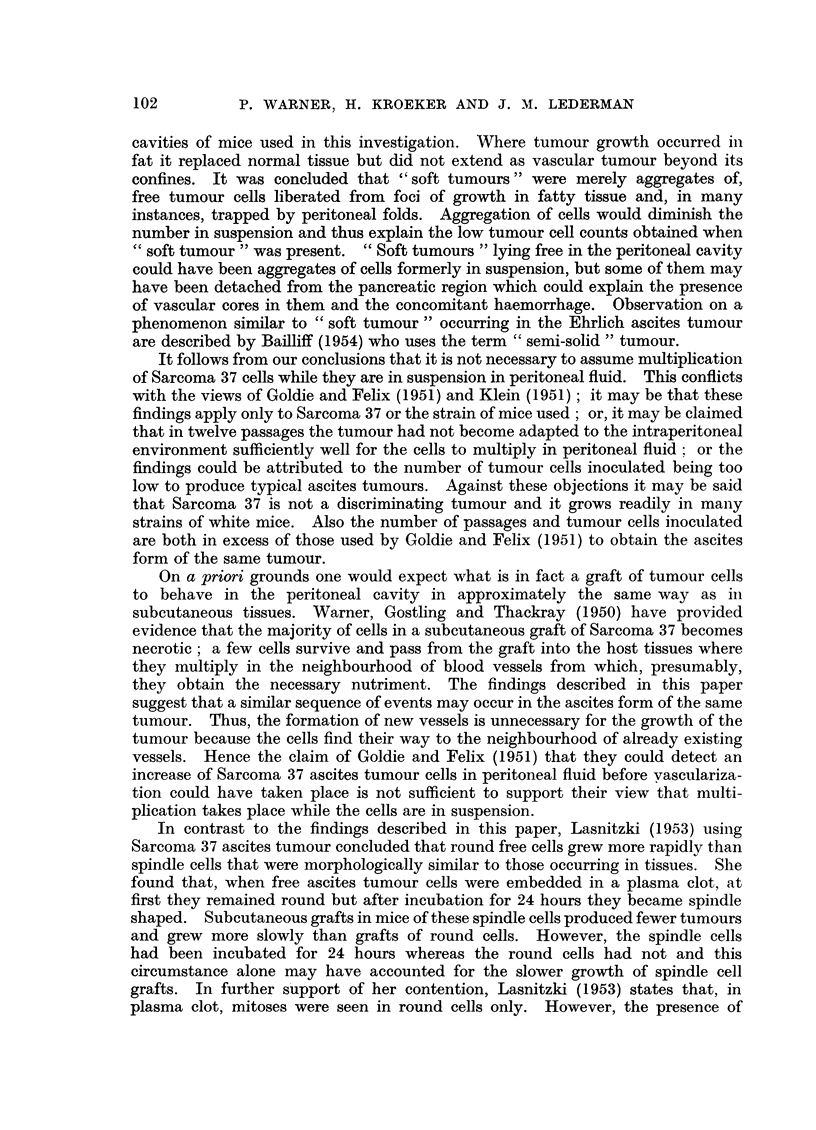

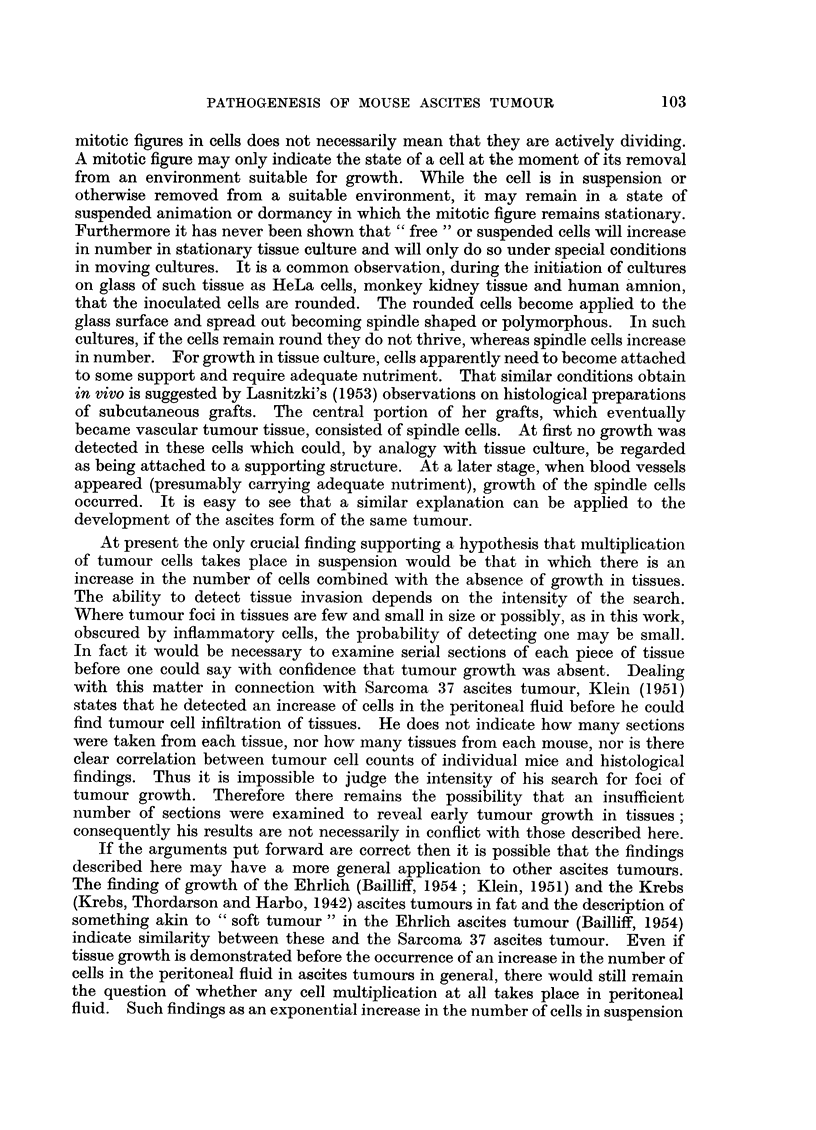

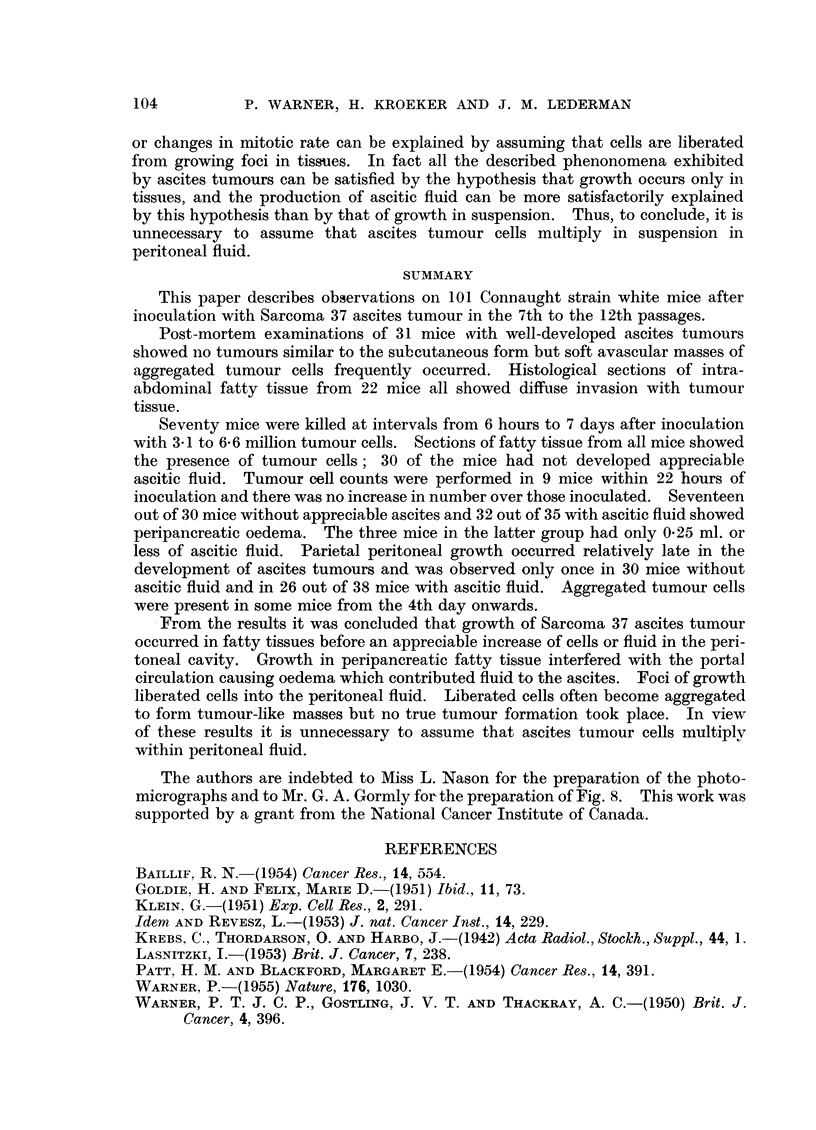


## References

[OCR_00798] BAILLIF R. N. (1954). The solid phase of the Ehrlich ascites tumor in mice.. Cancer Res.

[OCR_00803] KLEIN G., REVESZ L. (1953). Quantitative studies on the multiplication of neoplastic cells in vivo. I. Growth curves of the Ehrlich and MC1M ascites tumors.. J Natl Cancer Inst.

[OCR_00806] LASNITZKI I. (1953). The behaviour of ascites tumour cells in vitro and in vivo.. Br J Cancer.

[OCR_00808] PATT H. M., BLACKFORD M. E. (1954). Quantitative studies of the growth response of the Krebs ascites tumor.. Cancer Res.

[OCR_00811] WARNER P. T. J. C. P., GOSTLING J. V. T., THACKRAY A. C. (1950). The fate of grafts of sarcoma 37 mince after exposure to low temperature and freeze-drying.. Br J Cancer.

[OCR_00809] WARNER P. (1955). Growth of sarcoma 37 mouse ascites tumour in peritoneal fat.. Nature.

